# Dietary Cholesterol Is Highly Associated with Severity of Hyperlipidemia and Atherosclerotic Lesions in Heterozygous LDLR-Deficient Hamsters

**DOI:** 10.3390/ijms20143515

**Published:** 2019-07-18

**Authors:** Jinjie Wang, Kunxiang He, Chun Yang, Xiao Lin, Xin Zhang, Yuhui Wang, George Liu, Xunde Xian

**Affiliations:** 1 Institute of Cardiovascular Sciences and Key Laboratory of Molecular Cardiovascular Sciences, Ministry of Education, Peking University, Beijing 100191, China; 2 Hebei Invivo Biotech Co., Shijiazhuang 050000, China

**Keywords:** low-density lipoprotein receptor, familial hypercholesterolemia, hamster, dietary cholesterol, atherosclerosis

## Abstract

Objective: Familial hypercholesterolemia (FH) is a dominant inherited disease caused mainly by low-density lipoprotein receptor (LDLR) gene mutations. To different extents, both heterozygous and homozygous FH patients develop premature coronary heart disease (CHD). However, most of the experimental animal models with LDLR deficiency could not fully recapitulate FH because they develop hyperlipidemia and atherosclerosis only in homozygous, but not in heterozygous, form. In the current study, we investigated the responsiveness of the LDLR+/− hamster to dietary cholesterol and whether plasma cholesterol levels were positively associated with the severity of atherosclerosis. Approach and Methods: wild type WT and LDLR+/− hamsters were fed a high fat diet with different cholesterol contents (HCHF) for 12 or 16 weeks. Plasma lipids, (apo)lipoproteins, and atherosclerosis in both the aorta and coronary arteries were analyzed. After a HCHF diet challenge, the levels of total cholesterol (TC) in WT and LDLR+/− hamsters were significantly elevated, but the latter showed a more pronounced lipoprotein profile, with higher cholesterol levels that were positively correlated with dietary cholesterol contents. The LDLR+/− hamsters also showed accelerated atherosclerotic lesions in the aorta and coronary arteries, whereas only mild aortic lesions were observed in WT hamsters. Conclusions: Our findings demonstrate that, unlike other rodent animals, the levels of plasma cholesterol in hamsters can be significantly modulated by the intervention of dietary cholesterol, which were closely associated with severity of atherosclerosis in LDLR+/− hamsters, suggesting that the LDLR+/− hamster is an ideal animal model for FH and has great potential in the study of FH and atherosclerosis-related CHD.

## 1. Introduction

Familial hypercholesterolemia (FH) is characterized by elevated plasma low-density lipoprotein cholesterol (LDL-C) and coronary heart disease (CHD), which is largely caused by loss-of-function mutations in the *Ldlr* gene [1]. The incidence of heterozygous and homozygous FH in different ethnic populations is ~1/200 and 1/1,000,000, respectively, in which, to different extents, elevated LDL-C and coronary atherosclerosis are observed [2]. Because the heterozygous form is very prevalent in humans and significantly increases the risk of CHD, many heterozygous low-density lipoprotein receptor (LDLR) knockout (KO) animal models with different species are applied for the study of FH and CHD [3]. However, unlike clinical patients with heterozygous FH, no experimental heterozygous animals with LDLR deficiency displayed significant FH traits, in which hypercholesterolemia and atherosclerotic lesions cannot be elicited by environmental interventions, including diet [3]. Recently, Johnston et al. found that there was no association between plasma lipids and time-dependent atherosclerotic burden in different experimental murine models [4], thus limiting the applications of experimental animal models to the translational study of FH and drug screening, although they have been widely used for basic research.

Syrian golden hamsters are small rodent laboratory animals that are widely used in a variety of research fields, including oncology, virology, diabetes, and cardiovascular disease [5,6]. Unlike mice, which do not express cholesteryl ester transfer protein (CETP), a key protein required for lipid transfer in plasma, hamsters possess high CETP activity in plasma, like humans [7]. Furthermore, both humans and hamsters display apolipoprotein B (ApoB) editing only in the intestine, leading to ApoB48 only being present in intestinal-derived chylomicrons (CMs) and ApoB100-containing very-low-density lipoproteins (VLDLs) from the liver, which account for 80% of cholesterol synthesis through extracellular pathways [8]. Therefore, according to human-like metabolic features, hamsters are consistently sensitive to high-fat diets and are prone to hyperlipidemia and atherosclerosis by high-cholesterol/high-fat (HCHF) diet.

Previously, we successfully generated LDLR+/− hamsters using the CRISPR/CAS9 gene editing technique and found that LDLR+/− hamsters were predisposed to hypercholesterolemia and atherosclerosis compared to WT controls on HCHF diet [5]. In this study, we further explored whether plasma lipid levels could be modulated by dietary cholesterol and whether the levels of plasma lipid were closely associated with the severity of atherosclerotic lesions in the aorta and coronary arteries in WT and LDLR+/− hamsters. Our data will provide reliable evidence for the precise study of human FH and atherosclerosis-related CHD.

## 2. Results

### 2.1. Characteristics of Plasma Lipids, Apolipoproteins, and Lipoproteins in Wild Type (WT) and LDLR+/− Hamsters

**Figure 1:**
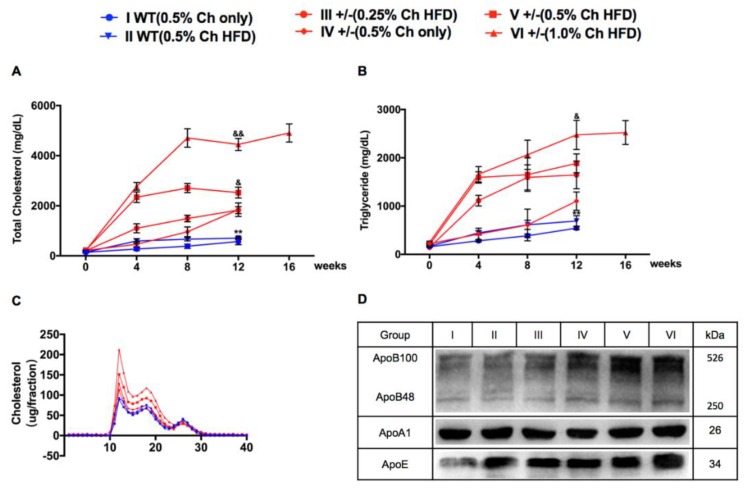
Plasma lipids and lipoprotein profiles in wild type (WT) and low-density lipoprotein receptor (LDLR)+/− hamsters with high-cholesterol (HC) or high-cholesterol/high-fat (HCHF) diet. (**A**,**B**) Plasma total cholesterol (**A**) and triglyceride (**B**) were measured at the indicated time points in WT and LDLR+/− hamsters on HC or HCHF diet for 12 or 16 weeks. *n* = 30/group. (**C**) Fast protein liquid chromatography (FPLC) analysis of 500 μL of pooled plasma lipoprotein profiles from WT and LDLR+/− hamsters. (**D**) Western blot analysis of plasma apolipoprotein (Apo) B, ApoA1, and ApoE levels from WT and LDLR+/− hamsters. ^&^ *p* < 0.05, ^&&^ *p* < 0.01 vs. group III, ** *p* < 0.01 vs. group IV.

To study the effects of dietary cholesterol and fat on hyperlipidemia and atherosclerosis in hamsters and the difference between WT and LDLR +/− hamsters, we first determined the lipid levels of hamsters before and after high-cholesterol (HC) or HCHF diets. We measured the basal value of plasma lipids two weeks before feeding the hamsters HC or HCHF diets and then measured the lipid levels every four weeks after feeding the hamsters HC or HCHF diets. Our results showed that the plasma lipid levels of WT and LDLR+/− hamsters were significantly increased upon adopting HC or HCHF diets. The total cholesterol (TC) and triglyceride (TG) levels of group I reached 138.2 ± 24.5 mg/dL and 586.0 ± 114.6 mg/dL, respectively, whereas the TC and TG levels of group II hamsters reached 143.6 ± 20.0 mg/dL and 693.0 ± 242.1 mg/dL, respectively (Figure 1A,B). Compared with WT hamsters, LDLR+/− hamsters showed a more pronounced increase in plasma lipid levels after 12-week high fat treatment. As shown in Figure 1, the TC levels in groups III–VI reached 1883.5 ± 395.6 mg/dL, 1841.2 ± 813.3 mg/dL, 2526.5 ± 609.5 mg/dL, and 4860.4 ± 935.3 mg/dL, respectively. The TG levels reached 1647.5 ± 857.6 mg/dL, 1273.3 ± 407.4 mg/dL, 2474.6 ± 850.5 mg/dL, and 3177.6 ± 2085.1 mg/dL, respectively. These results indicated that the plasma lipid levels of hamsters were positively correlated with the dietary cholesterol and fat contents. To better understand the plasma cholesterol distribution in WT and LDLR+/− hamsters, we performed fast protein liquid chromatography (FPLC) to analyze the lipoprotein in different fractions. The results showed that VLDL and LDL peaks of LDLR+/− hamsters were obviously higher than WT hamsters, and there was no difference in HDL peaks between WT and LDLR+/− hamsters (Figure 1C). Furthermore, we detected apolipoproteins levels in each group by Western blot. The results showed that ApoB100 was the major apolipoprotein in each group; however, ApoB and ApoE levels increased from group I to group VI, though ApoA1 levels did not alter over all groups (Figure 1D).

### 2.2. Atherosclerotic Development in LDLR+/− Hamsters Was Cholesterol Concentration and Time Course-Dependent

**Figure 2:**
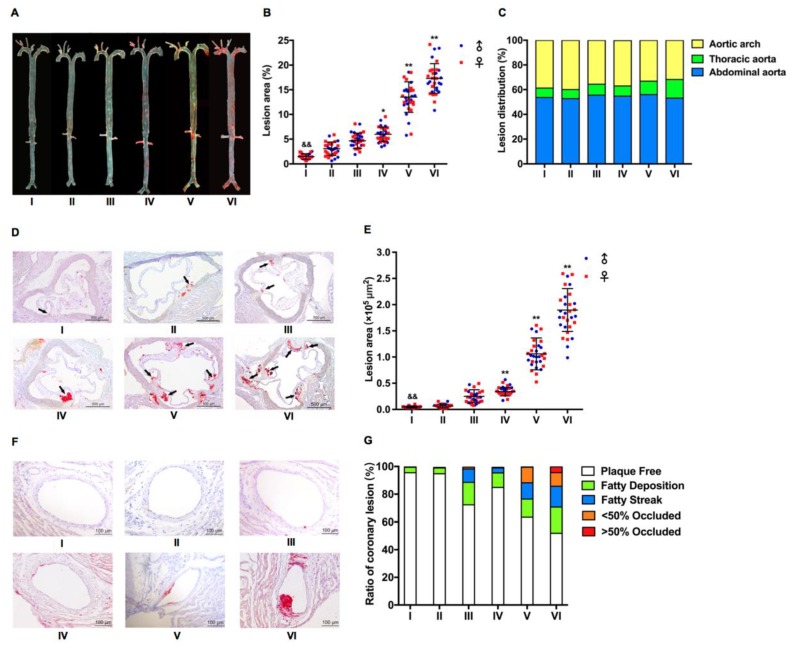
Characteristics of HC or HCHF diet-induced atherosclerosis in WT and LDLR+/− hamsters. (**A**) Representative en face images of Oil Red O stained whole aortas from WT and LDLR+/− hamsters on the HC or HCHF diets for 12 weeks as described above in Figure 1. (**B**) Lesion sizes in the whole aorta were quantified in the mixed-gender WT and LDLR+/− hamsters on the HC or HCHF diets for 12 weeks. *n* = 30/group. (**C**) Analysis of plaque distribution in WT and LDLR+/− hamsters on the HC or HCHF diets for 12 weeks. (**D**) Representative images of sectioned aortic roots with Oil Red O staining from WT and LDLR+/− hamsters on the HC or HCHF diets for 12 weeks as described above in Figure 1. Black arrows indicate Oil Red O positive staining. (**E**) Quantification of lesion areas in aortic roots. *n* = 30/group. (**F**) Representative images of coronary arteries stained with Oil Red O from WT and LDLR+/− hamsters on HC or HCHF diets for 12 weeks as described above in Figure 1. (**G**) Semi-quantification of atherosclerotic lesions in the coronary arteries of each group. *n* = 30/group. ^&&^ *p* < 0.01 vs. group III, * *p* < 0.05, ** *p* < 0.01 vs. group IV in (**B**,**E**).

It is to be noted that hyperlipidemia significantly elicits atherosclerosis, but on the regular chow diet, neither WT nor heterozygous hamsters developed atherosclerosis [5]. To investigate accelerated atherosclerosis and coronary artery disease in hamsters, we fed WT and LDLR+/− hamsters HC or HCHF diets. After 12 weeks of HC or HCHF diets, Oil Red O staining showed that atherosclerotic lesions were observed in both WT and LDLR+/− hamsters, especially in LDLR+/− hamsters. The statistical results showed that the proportion of aortic plaque in group I–VI hamsters was 1.6%, 3.1%, 4.5%, 6.2%, 13.5%, and 17.5%, respectively, but there was no difference between male and female hamsters. The lesion size of aortas was significantly increased in LDLR+/− hamsters, and the areas of aortic lesions were positively correlated with cholesterol contents in HF diet. The lesions in group V and VI were highly obvious after 12 weeks of HCHF diets (Figure 2A,B). In addition, we observed that the atherosclerotic plaques in the aorta were not evenly distributed. As shown in Figure 2A, the lesions were mainly restricted in the aortic arch and the abdominal aorta. Statistical analysis of lesion plaque distribution showed that the plaque areas of the aortic arch of group I–VI hamsters accounted for 38.7%, 39.9%, 35.6%, 36.8%, 33.1%, and 31.8% of the total plaque area, respectively, whereas the plaque area of the abdominal arch accounted for 53.7%, 52.8%, 55.5%, 54.9%, 56.0%, and 53.0% of the total plaque area, respectively. The aortic arch atherosclerotic lesions were most severely concentrated (Figure 2C).

**Figure 3:**
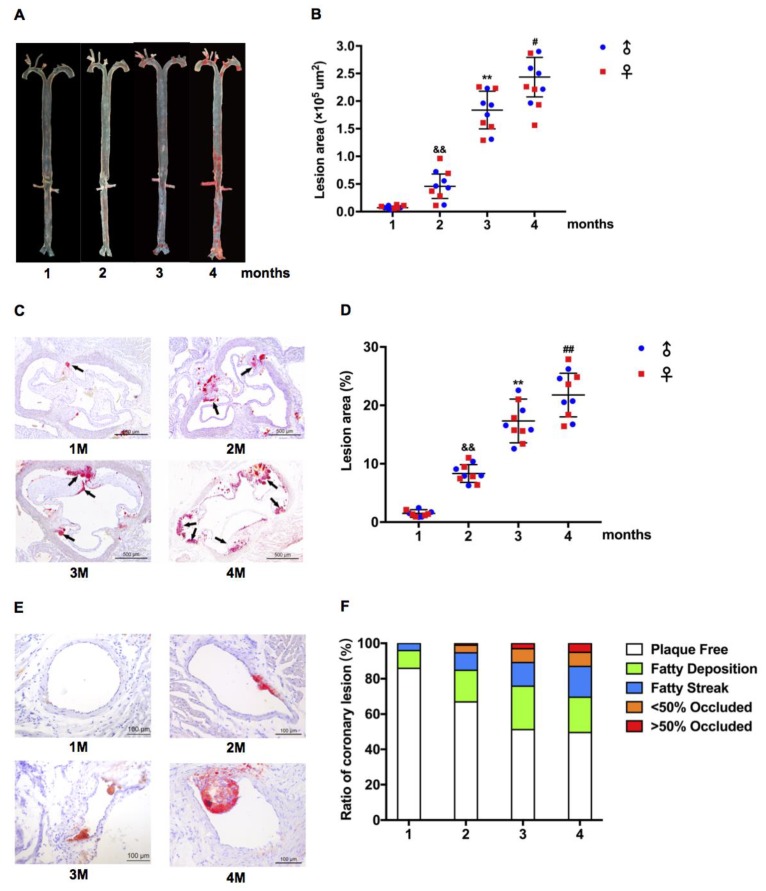
Time course analysis of atherosclerotic lesions in low-density lipoprotein receptor (LDLR)+/− hamsters on HCHF diet. (**A**) Characteristics of HCHF diet-induced atherosclerosis in LDLR+/− hamsters at indicated time points. (**B**) Lesion sizes in the whole aorta were quantified in the mixed-gender LDLR+/− hamsters on the HCHF diets for 1, 2, 3, and 4 months. *n* = 10/group. (**C**) Representative images of aortic root sections with Oil Red O staining from LDLR+/− hamsters on the HCHF diets for 1 (1M), 2 (2M), 3 (3M), and 4 (4M) months. (**D**) Quantification of lesion areas in aortic roots. *n* = 10/group. Black arrows indicate Oil Red O positive staining. (**E**) Representative images of coronary arteries stained with Oil Red O staining from LDLR+/− hamsters on the HCHF diets for 1 (1M), 2 (2M), 3 (3M), and 4 (4M). (**F**) Semi-quantification of atherosclerotic lesions in the coronary arteries of each group. *n* = 30/group. ^&&^ *p* < 0.01 vs. group 1, ** *p* < 0.01 vs. group 2, ^#^ *p* < 0.05, ^##^ *p* < 0.01 vs. group 3 in (**B**,**D**).

Next, we investigated the aortic root with Oil Red O staining and found that the area of atherosclerotic lesions of group I–VI hamsters was 5046 μm^2^, 6235 μm^2^, 25,060 μm^2^, 38,462 μm^2^, 105,965 μm^2^, and 190,840 μm^2^, respectively; however, there was no difference between males and females (Figure 2D,E). Coronary atherosclerosis is one of the traits of FH [5]. It was rational for us to determine the atherosclerosis in coronary artery and we found that the proportion of lesions in the coronary artery of group I–VI hamsters was 4.3%, 4.5%, 27.1%, 14.5%, 36.6%, and 47.8%, respectively (Figure 2F,G). Moreover, LDLR+/− hamsters were fed with HCHF diet containing 1.0% cholesterol and 15% fat for 4–16 weeks. The proportion of aortic plaque in group 1–4 months was 1.5%, 7.8%, 17.3%, and 21.8%, respectively (Figure 3A,B). The areas of atherosclerotic lesions of aortic root were 5612 μm^2^, 45,883 μm^2^, 183,686 μm^2^, and 243,473 μm^2^ (Figure 2C,D). The proportion of lesions in the coronary artery of group I–IV hamsters was 14.0%, 32.9%, 48.7%, and 50.3%, respectively (Figure 2E,F), suggesting that the atherosclerotic development in LDLR+/− was time course-dependent.

## 3. Discussion

Heterozygous FH is a relatively more common form, in which the incidence of CHD is increased by 20 folds. Because most of the heterozygous LDLR-deficient animal models do not significantly differ from wild-type controls, LDLR-deficient animal models with heterozygous form cannot be used to study FH. Previously, we found that HCHF-fed LDLR+/− hamsters with hypercholesterolemia and atherosclerotic lesions are more pronounced than the wild-type group, suggesting that severity of hypercholesterolemia and atherosclerosis may be modulated by diet intervention to achieve the experimental purpose of lipid disorder-related CHD.

In the present study, we showed that dietary cholesterol could significantly modulate the plasma concentration of TC and LDL-C, and the severity of atherosclerosis in LDLR+/− hamsters. Furthermore, we found that plasma cholesterol levels were positively correlated to the severity of atherosclerotic lesions in the aorta and coronary arteries, which indicates that circulating cholesterol, especially in LDL fraction, is a high risk factor of FH and atherosclerosis-related CHD.

Although LDLR-deficient mice, especially homozygous animals, have been widely used for human atherosclerosis, accumulated data have demonstrated that they cannot replicate FH traits and are not suitable for the translational study of lipid disorder-related CHD, because heterozygotes are completely healthy and homozygotes exhibit mildly elevated cholesterol levels on regular chow diet [9]. However, after a change with HFHC diet containing 0.5% cholate (Paigen diet), only homozygous mice showed hypercholesterolemia and atherosclerosis, whereas heterozygous animals were still resistant to diet-induced FH phenotype. Additionally, independent research groups reported the limitations of murine models of studying human atherosclerosis, implying that more species should be considered [10,11]. Recently, a meta-analysis study demonstrated that there is no significant correlation of degree of dyslipidemia and the burden of atherosclerosis in murine animals, including homozygous LDLR-deficient mice [4].

Interestingly, Dillard et al. found that wild-type hamsters have many advantages in the field of lipid metabolism; however, the atherosclerotic development in response to diet intervention is inconsistent in the hamsters regardless of the strains, suggesting that wild-type hamsters are not considered to be a useful animal model for studying high fat diet-induced atherosclerosis [12]. In contrast to LDLR-deficient mice and wild-type hamsters, the degree of hypercholesterolemia can be easily modulated by dietary cholesterol in LDLR+/− hamsters, in which the severity of atherosclerotic lesions is closely associated with plasma cholesterol concentration. Moreover, the atherosclerotic distribution in HFHC diet-fed LDLR+/− hamsters is similar to that observed in heterozygous FH that has not been seen in murine animals.

In conclusion, we demonstrate for the first time that plasma cholesterol in LDLR+/− hamsters can be significantly modulated by dietary cholesterol, which was highly associated with severity of atherosclerosis in the aorta and coronary arteries, suggesting that, compared to other heterozygous animal models with LDLR deficiency, the LDLR+/− hamster will display a great advantage in the study of diet-induced hyperlipidemia, atherosclerosis-related CHD, and the high-throughput screening of drugs targeting LDLR in future.

## 4. Materials and Methods

### 4.1. Animals

Syrian golden hamsters were purchased from Vital River Laboratories (Beijing, China) and maintained in a specific-pathogen-free (SPF) condition with a 14/10-h light/dark cycle. In our studies, male and female animals of 8–12 weeks of age were used. WT hamsters were fed with a high-cholesterol diet containing 0.5% cholesterol only (group I) and an HFHC diet containing 0.5% cholesterol and 15% fat (group II). The LDLR+/− hamsters were fed with HFHC diet containing 0.25% cholesterol and 15% fat (group III), high-cholesterol diet containing 0.5% cholesterol only (group IV), HFHC diet containing 0.5% cholesterol and 15% fat (group V), or HFHC diet containing 1.0% cholesterol and 15% fat (group VI) for 12 weeks. In the meantime, LDLR+/− hamsters were fed with HFHC diet containing 1.0% cholesterol and 15% fat for 4 (group 1), 8 (group 2), 12 (group 3), and 16 weeks (group 4), respectively. Plasma lipids were tested every 4 weeks. All experiments were performed under the principle of experimental animal health (NIH released no.85Y231996 Revision) and approved by the laboratory animal ethics committee of Peking University (LA2010-059; 15 March 2010).

### 4.2. Analysis of Plasma Lipids and (apo)Lipoproteins

Plasma was collected from the hamsters after 12 h fasting, and TC and TG levels were determined using commercially available kits (MAK043 and TR0100, Sigma, Saint Louis, MO, USA). Western blots were used to detect plasma ApoA1, ApoE, and ApoB. Briefly, 1 μL plasma was mixed with buffer containing sodium dodecyl sulfate (SDS) and dithiothreitol (DTT), and boiled at 95 °C for 10 min. The samples were loaded to 6% or 12% sodium dodecyl sulfate polyacrylamide gel (SDS-PAGE) for ApoB or ApoA1 and ApoE. The following antibodies were used: ApoA-I (ABS443, Millipore, San Diego, CA, USA, rabbit polyclonal IgG, 1:5000), ApoE (178479, Millipore, goat polyclonal IgG, 1:5000), and ApoB (178467, Millipore, goat polyclonal IgG, 1:5000). Plasma lipoprotein profiles were analyzed by FPLC. The pooled plasma (250 μL) of each group was applied to Tricorn high-performance Superose S-6 10/300GL column (Amersham Biosciences, Little Chalfont, Buckinghamshire, UK), followed by an elution with PBS at a constant flow rate of 0.25 mL/min. The TC and TG levels in each fraction (500 μL) were determined using the same cholesterol and TG kits.

### 4.3. Pathological Analysis

The WT and LDLR+/− hamsters were fed with high-cholesterol diet or different kinds of HCHF diet for 12 weeks, LDLR+/− hamsters were fed with HCHF diet containing 1.0% cholesterol, 15% fat for 16 weeks. Then the animals were sacrificed and perfused with 20 mL of 0.01 M PBS through the left ventricle. Hearts, whole aortas, and other tissues were placed in 4% paraformaldehyde solution for 1 week and transferred to 20% sucrose solution overnight. Hearts were fixed at Optimal Cutting Temperature (OCT) and cross-sectioned (7 μm per slice). The atherosclerotic plaques in whole aortas (en face) and aortic roots were analyzed by staining with a 0.3% Oil Red O solution (Sigma-Aldrich, St. Louis, MO, USA) for 30 min, followed by scrubbing with 60% isopropanol and distilled water. The whole aorta was divided into three parts as defined previously [1]: (1) The aortic arch: Aortic root to 3 mm below the left subclavian; (2) the thoracic aorta: The region between the end of the arch and the last intercostal branch; (3) the abdominal aorta: The region between the end of thoracic aorta segment and the iliac bifurcation. To analyze the atherosclerotic plaques, coronary atherosclerosis was scored by the plaques containing fatty streaks and was expressed as the proportion of the total coronary arteries, which were plaque free, <5% fatty deposition, fatty streak, occluded, <50% occluded, and >50% occluded.

### 4.4. Statistical Analysis

The data were analyzed by Student’s *t*-test or two-way analysis of variance (ANOVA). All *p*-values less than 0.05 were considered statistically significant. All data were expressed as mean ± SEM.
